# G9a inactivation in progenitor cells with Isl1-Cre with reduced recombinase activity models aspects of Dandy-Walker complex

**DOI:** 10.1242/bio.059894

**Published:** 2023-07-28

**Authors:** Lijun Chi, Ling Zhong, Dorothy Lee, Xinwen Yu, Amalia Caballero, Brian Nieman, Paul Delgado-Olguin

**Affiliations:** ^1^ Translational Medicine, The Hospital for Sick Children, Toronto, ON M5G0A4, Canada; ^2^ Department of Endocrinology, National Health Committee Key Laboratory of Endocrinology, Peking Union Medical College Hospital, Chinese Academy of Medical Sciences and Peking Union Medical College, Beijing 100730, China; ^3^ Department of Physiology, University of Toronto, Toronto, ON M5S1A8, Canada; ^4^ Department of Molecular Genetics, University of Toronto, Toronto, ON M5S1A8, Canada; ^5^ Mouse Imaging Centre (MICe), The Hospital for Sick Children, Toronto, ON M5T3H7, Canada; ^6^ Ontario Institute for Cancer Research, Toronto, ON M5G0A3, Canada; ^7^ Department of Medical Biophysics, University of Toronto, Toronto, ON M5G1L7, Canada; ^8^ Heart and Stroke Richard Lewar Centre of Excellence, Toronto, ON M5S3H2, Canada

**Keywords:** Dandy-Walker complex, G9a, *Isl1*-expressing progenitors, Neural crest development, Progenitor cell development

## Abstract

G9a, also known as EHMT2, is essential for embryogenesis and has specific functions in multiple developmental processes. G9a inactivation affects development of the nervous system, which is formed with contribution of descendants of progenitor cells expressing the transcription factor Isl1. However, the function of G9a in *Isl1*-expressing progenitors is unknown. Here, we show that G9a is required for proper development of multiple structures formed with contribution of *Isl1*-expressing progenitors. A Cre-dependent *GFP* reporter revealed that the recombinase activity of the Isl1-Cre used in this study to inactivate G9a was reduced to a subset of *Isl1*-expressing progenitor cells. *G9a* mutants reached endpoint by 7 weeks of age with cardiac hypertrophy, hydrocephalus, underdeveloped cerebellum and hind limb paralysis, modeling aspects of Dandy-Walker complex. Moreover, neuroepithelium of the lateral ventricle derived from *Isl1*-expressing progenitors was thinner and disorganized, potentially compromising cerebrospinal fluid dynamics in *G9a* mutants. Micro-computed tomography after iodine staining revealed increased volume of the heart, eye lens and brain structures in *G9a* mutant fetuses. Thus, altered development of descendants of the second heart field and the neural crest could contribute to multicomponent malformation like Dandy-Walker.

## Teaser

**Summary:** Analysis of the effect of conditional G9a inactivation mediated by Isl1-Cre with decreased recombinase activity in mice indicated that dysregulation of G9a-controlled gene expression contributes to Dandy-Walker complex.

## INTRODUCTION

G9a, a histone methyltransferase also known as euchromatic histone-lysine N-methyltransferase 2 (EHMT2), di-methylates the lysine 9 of histone H3 (H3K9me2) (Tachibana et al., 2001). G9a cooperates in stoichiometric complex with its closely related homolog EHMT1, also known as GLP, to deposit H3K9me2 in euchromatin (Tachibana et al., 2005) predominantly in repressed gene promoters. H3K9me2 also mediates heterochromatin establishment and silencing of repetitive elements and transposons (Padeken et al., 2022; Tachibana et al., 2002). Although G9a is predominantly associated with gene repression, it is required for the proper activation of specific differentiation programs. H3K9me contributes to silencing differentiation drivers until a cell fate decision program is activated, then contributes to silencing the progenitor stage program (Padeken et al., 2022). Accordingly, G9a regulates lineage segregation in the blastocyst (Zylicz et al., 2018) and is essential for mammalian development. Its constitutive deficiency in mice causes lethality between embryonic day (E)9.5 and E12.5 (Tachibana et al., 2002). Moreover, G9a has specific functions in multiple developmental processes. For example, G9a in endothelial cells controls maturation of the placental vasculature (Chi et al., 2017), and in retinal progenitors suppresses the proliferative state to promote differentiation and development of the retina (Katoh et al., 2012). G9a has a dual function in the heart; it prevents cardiac hypertrophy at baseline but promotes it in response to pathological stress (Papait et al., 2017). In neural development *in vitro*, G9a regulates proliferation of neural progenitors (Hou et al., 2021), neurogenesis (Fiszbein et al., 2016; Kim et al., 2016; Olsen et al., 2016), and neurite (Fiszbein et al., 2016) and axon growth (Wilson et al., 2020). In the postnatal brain, G9a regulates neurocognition (Schaefer et al., 2009). The broad effects of G9a inactivation suggest that its dysfunction could contribute to multicomponent diseases by affecting the development of multiple progenitor cell derivatives.

Expression of the LIM homeodomain transcription factor islet 1 (*Isl1*) in the mammalian embryo defines a cell population composed of pluripotent progenitors of the lateral splanchnic mesoderm, a subpopulation of neural crest progenitors that express *Wnt1* (Engleka et al., 2012) and a subset of hind limb mesenchyme progenitors (Yang et al., 2006). A subgroup of *Isl1*-expressing progenitors of the lateral splanchnic mesoderm gives rise to the second heart field (Nathan et al., 2008), which differentiates into cardiomyocytes of the right ventricle, and the outflow tract. These progenitors also contribute portions of cardiac endothelium, smooth muscle and atrial cardiomyocytes (Cai et al., 2003; Moretti et al., 2006). Another subgroup of *Isl1*-positive progenitors of lateral splanchnic mesoderm contributes to the formation of craniofacial muscles (Nathan et al., 2008). The neural crest progenitor subpopulation expressing *Isl1* known as the cardiac neural crest migrates from the cranial neural tube to contribute to portions of the outflow tract, valves and major arteries of the heart (George et al., 2020). The relevance of these progenitors is underscored by the fact that second heart field derivatives do not develop (Cai et al., 2003), hind limb development is abnormal (Narkis et al., 2012), and differentiation of neurons and other cell types in the neural tube is deficient in *Isl1* knockout mice (Pfaff et al., 1996), which die at E10 (Cai et al., 2003). Moreover, defective development of second heart field (Bruneau, 2008) and neural crest (Takahashi et al., 2013) derivatives is implicated in congenital disease. For example, Dandy-Walker, a rare syndrome affecting 1 in 30,000 births, presents with central nervous system and craniofacial defects (Coban et al., 2010) associated with heart (27% of patients) (Coban et al., 2010; Huong et al., 1975; Olson et al., 1981; Stambolliu et al., 2017) and limb (Sasaki-Adams et al., 2008; Stevens and Lachman, 2010) malformation. The characteristic central nervous system abnormality is cerebellar hypoplasia, with dilation of the fourth ventricle, enlargement of the posterior cranial fossa and hydrocephalus (Di Nora et al., 2022). Associated cardiac defects include incomplete atrial and ventricular septation (Coban et al., 2010; Huong et al., 1975; Olson et al., 1981), and cardiac hypertrophy that progressed to heart failure was reported in one case (Kurdi et al., 2009). Limb skeleton dysplasia and mesomelic shortening have also been reported (Stevens and Lachman, 2010). The co-occurrence of such variations led to the hypothesis that altered development and migration of neural crest cells contributes to Dandy-Walker (Coban et al., 2010; Squires et al., 1998). Genes regulating neural crest development, including Zic family member 1 and 4 (*ZIC1*, *ZIC4*) (Blank et al., 2011), fibroblast growth factor 8 and 17 (*FGF8*, *FGF17*) (Zanni et al., 2011), laminin subunit gamma 1(*LAMC1*) (Darbro et al., 2013), forkhead box C1 (*FOXC1*) (Aldinger et al., 2009), forkhead box L2 (*FOXL2*) (Lim et al., 2011) and cellular inhibitor of PP2A (*CIP2A*) (Yang et al., 2018), are mutated in Dandy-Walker. However, the contribution of abnormal neural crest progenitor development to Dandy-Walker complex has not been tested.

Here, we analyzed the effect of conditional *G9a* inactivation mediated by Isl1-Cre with decreased recombinase activity and observed that it models components of Dandy-Walker complex in mice.

## RESULTS

### Decreased recombinase activity of Isl1-Cre in transgenic mice

**Fig. 1.**
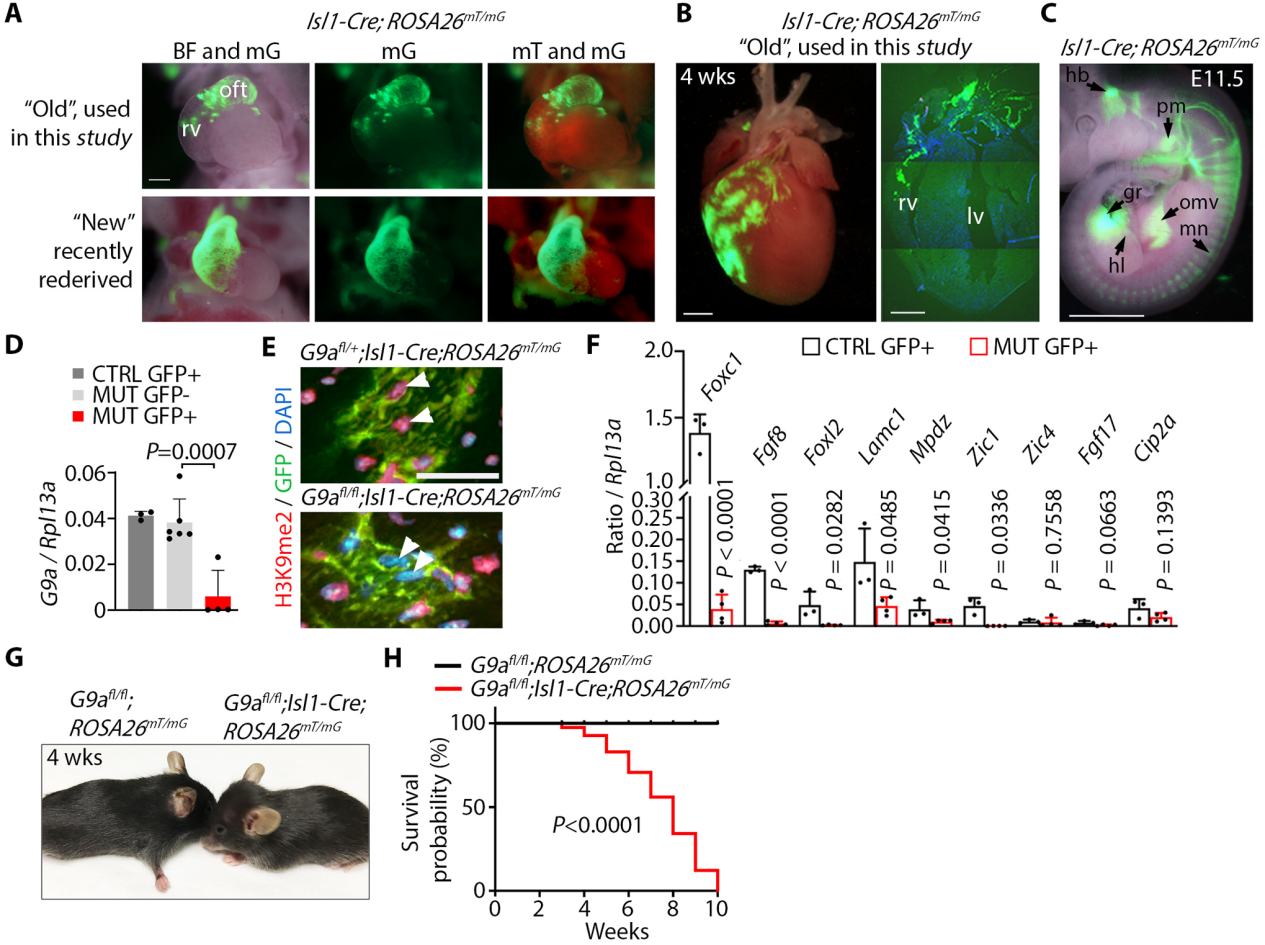
**G9a is required in a subset of *Isl1*-expressing progenitor cells for survival.** (A) Frontal views of hearts of E10.5 embryos carrying the ‘old’ *Isl1-Cre* and the Cre reporter *ROSA26^mT/mG^* used in this study compared to a recently rederived ‘new’ mouse line. Images were taken in bright field (BF) overlapped with membrane GFP (mG), and mG overlapped with membrane Tomato (mT). oft, outflow tract; rv, right ventricle. Scale bar: 250 µm. (B) GFP fluorescence in a whole heart (left), and GFP immunofluorescence in a section of a 4-week-old whole heart (right), from a mouse carrying *Isl1-Cre;ROSA26^mT/mG^*. lv, left ventricle. Scale bars: 2 mm. (C) GFP fluorescence in an *Isl1-Cre;ROSA26^mT/mG^* E11.5 embryo showing signal in the hindbrain (hb), pharyngeal mesoderm (pm), omphalomesenteric vessels (omv), motor neurons (mn), hind limb (hl) and the gonadal region (gr). Scale bar: 1 mm. (D) qPCR of *G9a* relative to *Rpl13a* on GFP-positive (GFP+) and GFP-negative cells (GFP−) sorted from control *G9a* heterozygote (*G9a^fl/+^*;*Isl1-Cre;ROSA26^mT/mG^*) and mutant (*G9a^fl/fl^*;*Isl1-Cre;ROSA26^mT/mG^*) E11.5 embryos. Bars represent the mean±s.d. of three, six and four embryos from control (CTRL) GFP+, mutant (MUT) GFP− and MUT GFP+, respectively. Data were analyzed by paired two-tailed Student's *t*-test. (E) Immunofluorescence of H3K9me2 and GFP in sections of control and mutant 4-week-old hearts. Nuclei were counterstained with DAPI. Arrowheads point to nuclei of GFP+ cells with decreased H3K9me2. Scale bar: 100 µm. (F) qPCR of *Foxc1*, *Fgf8*, *Foxl2*, *Lamc1*, *Mpdz*, *Zic1*, *Zic4*, *Fgf17* and *Cip2a*, relative to *Rpl13a*, on GFP+ cells sorted from control (*G9a^fl/+^*;*Isl1-Cre;ROSA26^mT/mG^*) or *G9a* mutant E11.5 embryos. Bars represent the mean±s.d. of cells sorted from three and four embryos per genotype. Data were analyzed by paired two-tailed Student's *t*-test. (G) *G9a* mutant mice (*G9a^fl/fl^*;*Isl1-Cre;ROSA26^mT/mG^*, from the ‘old’ Cre line) appear smaller than controls (*G9a^fl/fl^;ROSA26^mT/mG^*) and developed a domed head. (H) Survival curve of control and mutant mice analyzed by log-rank (Mantel–Cox) test. *n*=31 control and 27 mutant mice.

Conditional gene inactivation in which *Isl1-Cre* mice were crossed with multiple lines carrying ‘floxed’ alleles suddenly began producing fewer homozygous mutant offspring presenting phenotypes that were otherwise highly penetrant (data not shown). This suggested decreased gene inactivation efficiency, which can happen with an increased number of generations of Cre transgenic lines (Schulz et al., 2007). To test the recombination efficiency of Isl1-Cre, we crossed *Isl1-Cre* males with *ROSA26^mT/mG^* mice. *ROSA26^mT/mG^* constitutively expresses membrane-Tomato and membrane-GFP upon Cre-mediated recombination (Muzumdar et al., 2007). GFP fluorescence in embryos derived from this cross revealed that the recombination activity of Cre in the mouse line used in this study (‘old’ *Isl1-Cre*) was decreased compared to that in a freshly rederived *Isl1-Cre* line (‘new’ *Isl1-Cre*) (Fig. 1A). *ROSA26^mT/mG^* embryos and 4-week-old mice carrying the old *Isl1-Cre* expressed GFP in a smaller population of second heart field progenitors mostly contributing to the outflow tract and patches of cells in the right ventricle (Fig. 1A,B). In contrast, the new *Isl1-Cre* induced recombination in progenitors contributing to the entire outflow tract and right ventricle (Fig. 1A). GFP fluorescence revealed that the old Isl1-Cre was active in embryonic derivatives of *Isl1*-expressing progenitors (Zhuang et al., 2013), including the hindbrain, pharyngeal mesoderm, omphalomesenteric vessels, spinal motor neurons, gonadal region and the hind limb (Fig. 1C). This suggests that although the old *Isl1-Cre* transgene labeled multipotent *Isl1-*expressing progenitor cells, its recombination activity was limited to a subset of these progenitors.

### G9a is essential for development of descendants of *Isl1*-expressing progenitors

To determine the requirement of G9a in development of descendants of *Isl1*-expressing progenitors, we inactivated G9a via Isl1-Cre-mediated homologous recombination in mice. Males carrying a heterozygous *G9a* ‘floxed’ allele (Sampath et al., 2007) and the *Isl1-Cre* transgene (*G9a^fl/+^;Isl1-Cre*) were crossed with *G9a^fl/fl^;ROSA26^mT/mG^* females. When using the new *Isl1-Cre* line, *G9a* mutants (*G9a^fl/fl^;Isl1-Cre;ROSA26^mT/mG^*) died during embryogenesis, precluding analysis of the postnatal consequences of *G9a* deletion. In this study, we describe the effect of inactivating G9a using the old *Isl1-Cre* line*.* Mutant offspring of males carrying the old *Isl1-Cre* crossed with *G9a^fl/fl^;ROSA26^mT/mG^* females were born at the expected Mendelian ratio. We assessed G9a inactivation by quantitative PCR (qPCR) on cells isolated by fluorescence-activated cell sorting from embryos at day 11.5 of development (E11.5). *G9a* was ablated in mutant GFP-positive, but not in wild-type GFP-positive or mutant GFP-negative, cells (Fig. 1D). Moreover, H3K9me2 was decreased in nuclei of GFP-positive cells in 4-week-old hearts of *G9a* homozygous mutants, but not heterozygous mutants, as shown by immunofluorescence (Fig. 1E). This indicates that the old Isl1-Cre inactivated G9a in cells in which *ROSA26^mT/mG^* was recombined. To assess the function of G9a in gene regulation, we performed qPCR of neural crest development regulators. Whereas *Zic4* (Blank et al., 2011), *Fgf17* (Xu et al., 2000) and *Cip2a* were unaffected, *Foxc1* (Haldipur et al., 2014; Seo and Kume, 2006), *Fgf8* (Creuzet et al., 2004; Xu et al., 2000), *Foxl2* (Heude et al., 2015), *Lamc1* (Cao et al., 2007), *Mpdz* (Feldner et al., 2017) and *Zic1* (Plouhinec et al., 2014) were downregulated in GFP-positive cells sorted from E11.5 *G9a* mutant, compared to cells of control embryos (Fig. 1F). These genes are mutated in people affected by Dandy-Walker. Thus, G9a is required for proper expression of neural crest development regulators associated with Dandy-Walker in *Isl1*-expressing progenitor cells.

*G9a* heterozygous mutant mice were indistinguishable from *G9a^fl/fl^* controls (data not shown), but homozygous mutants were smaller and developed a domed head apparent by 3 weeks of age (Fig. 1G). Moreover, despite the hind limb skeleton appearing normal at 4 weeks (Fig. S1), *G9a* mutants lost hind limb mobility at 3 weeks (Movies 1 and 2) and reached endpoint at a median age of 7 weeks (Fig. 1H). This suggests an essential function of G9a in the development of multiple derivatives of *Isl1*-expressing progenitor cells.

### G9a inactivation in a subset of second heart field progenitors causes cardiac hypertrophy

**Fig. 2.**
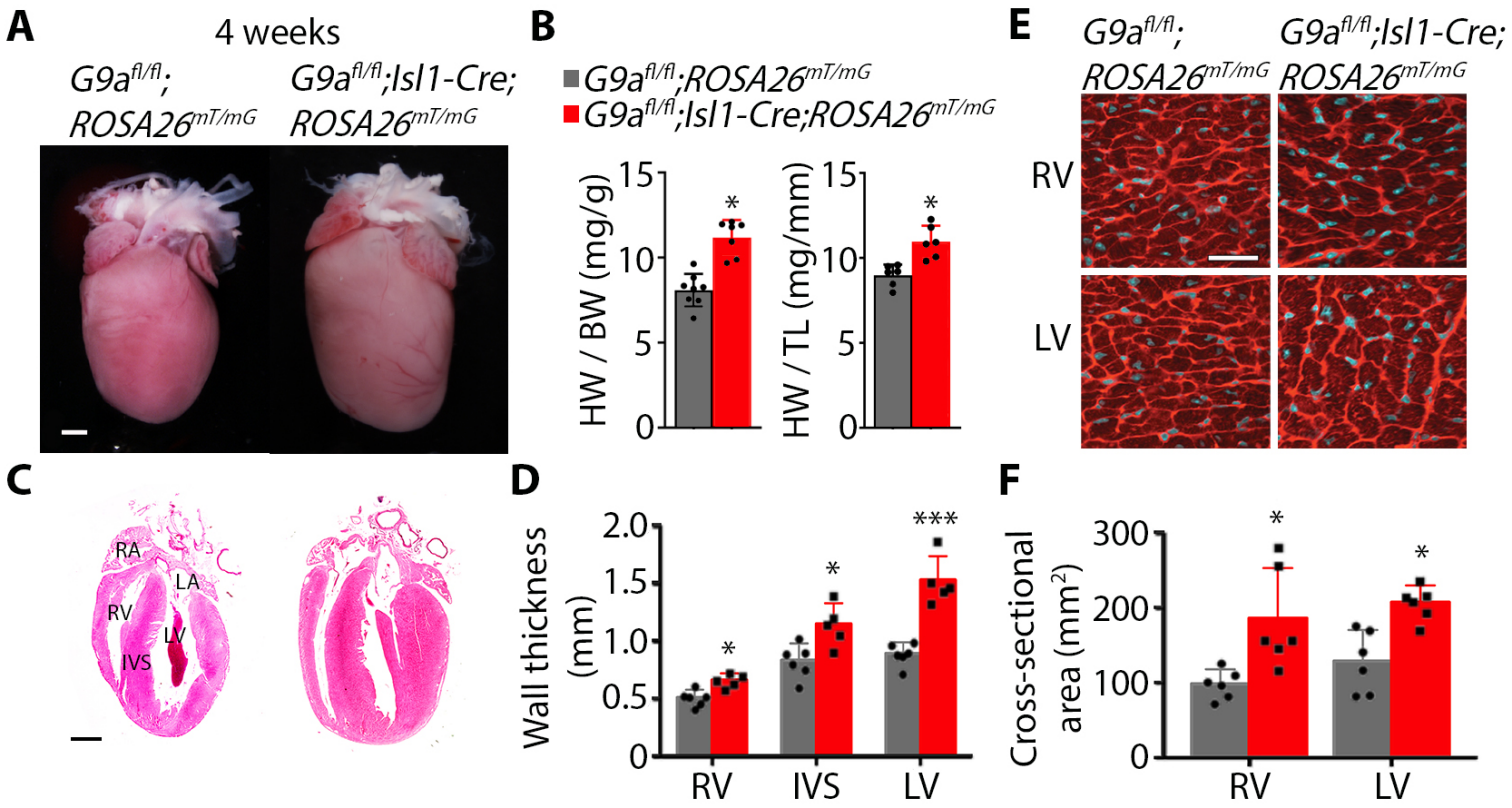
**G9a inactivation in *Isl1*-expressing progenitors causes cardiac hypertrophy.** (A) Hearts of 4-week-old control (*G9a^fl/fl^;ROSA26^mT/mG^*) and *G9a* mutant (*G9a^fl/fl^*;*Isl1-Cre;ROSA26^mT/mG^*) mice. Scale bar: 1 mm. (B) Heart weight (HW) to tibia length (TL), and heart weight to body weight (BW), ratios of control and mutant mice. Bars are the mean±s.d. Two-tailed Student's *t*-test. *P<0.05. *n*=6 hearts per group. (C) Coronal heart sections of control and mutant mice stained with Hematoxylin and Eosin. RA, right atrium; LA, left atrium; RV, right ventricle; IVS, interventricular septum; LV, left ventricle. Scale bar: 1 mm. (D) Thickness of the RV, IVS and LV measured from histological sections. Bars are the mean±s.d. Two-tailed Student's *t*-test. *P<0.05, ***P<0.001. *n*=6 control and 5 mutant hearts. (E) Wheat germ agglutinin staining on sections of the RV and LV of control and mutant hearts. Scale bar: 25 µm. (F) Cross-sectional area of cardiomyocytes of the RV and LV measured from sections stained with wheat germ agglutinin. Bars are the mean±s.d. Two-tailed Student's *t*-test. *P<0.05. *n*=5 control and 6 mutant hearts.

*Isl1*-expressing progenitor cells of the second heart field contribute to the heart's outflow tract, right ventricle and endocardial cushions required for cardiac septation. To assess the requirement for G9a in development of the heart, we analyzed heart morphology in *G9a^fl/fl^;Isl1-Cre;ROSA26^mT/mG^* mutants. The outflow tract, interventricular septum and overall cardiac morphology were normal in 4-week-old mutants. However, mutant hearts appeared bigger than control hearts (Fig. 2A) despite mutant mice being smaller (Fig. 1G). Accordingly, the heart weight to tibia length and heart weight to body weight ratios were higher (Fig. 2B), and the right and left ventricular wall and the interventricular septum were thicker, in the mutant mice than in *G9a^fl/fl^;ROSA26^mT/mG^* controls (Fig. 2C,D). Moreover, cardiomyocyte cell surface area was increased in mutants, as shown in heart sections stained with wheat germ agglutinin to outline cell membranes (Fig. 2E,F). This suggests that deficiency of G9a in second heart field progenitors does not grossly alter cardiac development but leads to cardiac hypertrophy in adulthood.

### Deficiency of G9a in neural crest progenitors expressing *Isl1* causes hydrocephalus

**Fig. 3.**
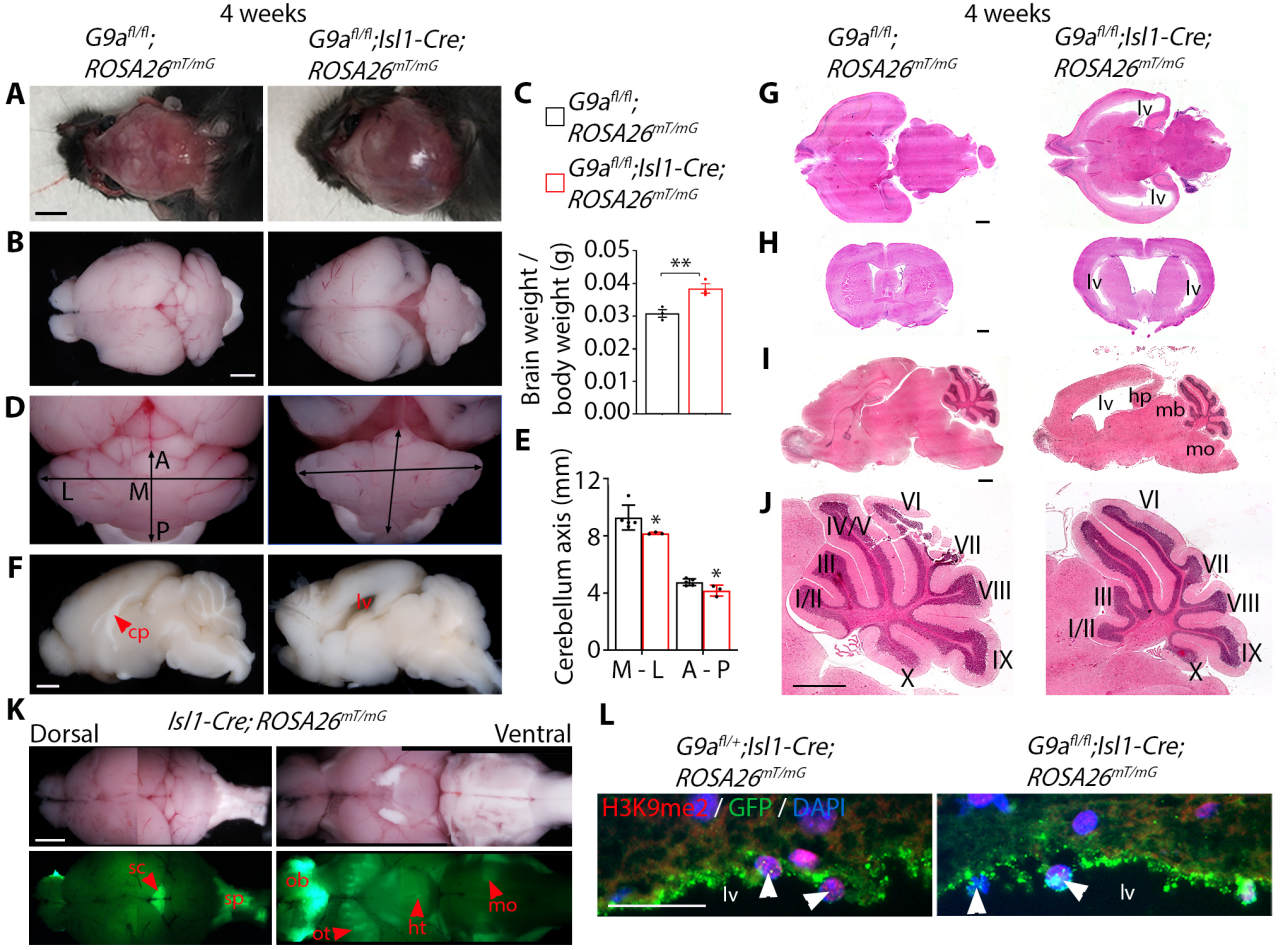
**G9a deficiency in *Isl1*-expressing progenitors causes hydrocephalus.** (A) Dorsal view of the partially dissected skull of 4-week-old control (*G9a^fl/fl^;ROSA26^mT/mG^*) and *G9a* mutant (*G9a^fl/fl^*;*Isl1-Cre;ROSA26^mT/mG^*) mice. Scale bar: 5 mm. (B) Dorsal view of the brain of control and mutant mice. Scale bar: 1 mm. (C) Brain weight to body weight ratio. Bars are the mean±s.d. Two-tailed Student's *t*-test. **P<0.01. *n*=3 control and 3 mutant mice. (D) Dorsal view of the cerebellum of control and *G9a* mutant mice. Arrows indicate the mediolateral (M and L) and anteroposterior (A and P) axes. (E) Length of the mediolateral (M-L) and anteroposterior (A-P) cerebellar axes. Bars are the mean±s.d. Two-tailed Student's *t*-test. *P<0.05. (F) Midsagittal view of the right side of fixed control and mutant brains. cp, corpus callosum; lv, lateral ventricle. Scale bar: 1 mm. (G,H) Transverse (G) and coronal (H) sections, stained with Hematoxylin and Eosin, of the brain of control and mutant mice. Scale bars: 1 mm. (I) Midsagittal section, stained with Hematoxylin and Eosin, of the brain of control and mutant mice. hp, hippocampus; mb, midbrain; mo, medulla oblongata. Scale bar: 1 mm. (J) Cerebellum close up of the sections in I. Cerebellar folds are indicated in Roman numerals. Scale bar: 1 mm. (K) Bright-field and GFP fluorescence of dorsal and ventral views of whole brains of 4-week-old *Isl1-Cre;ROSA26^mT/mG^* mice showing fluorescence in the superior colliculus (sc), spinal cord (sp), olfactory bulb (ob), and clusters of cells in the olfactory tubercule (ot), hypothalamus (ht) and medulla oblongata (mo). Scale bar: 1 mm. (L) H3K9me2 and GFP immunofluorescence in brain sections of control and mutant mice showing GFP+ neuroendothelial cells lining the lateral ventricle (lv). Arrowheads point to nuclei of GFP+ cells. Scale bar: 25 µm.

G9a has important functions in cardiac and neural descendants of *Isl1*-expressing progenitor cells (Deimling et al., 2017; Inagawa et al., 2013). *G9a* mutants developed a domed head due to subdural hematoma (Fig. 3A). The whole brain appeared bigger in *G9a* mutants than in controls; accordingly, the total brain mass was higher (Fig. 3B,C). The vermis and hemispheres were defined in mutant cerebella; however, the cerebellum was smaller than in controls as shown by shorter mediolateral and anteroposterior axes (Fig. 3D,E). The corpus callosum also appeared underdeveloped, and the lateral ventricle was larger in *G9a* mutants (Fig. 3F). The cerebral cortex of the right and left hemispheres towards the cerebellum appeared thinner in mutants than in controls (Fig. 3B), and histological analysis revealed enlarged lateral ventricles, indicative of hydrocephalus (Fig. 3G-I). As a result of fluid accumulation in the lateral ventricles, the hippocampus was displaced caudally, and the midbrain ventrally and caudally, pressing against cerebellar folds I/II and III, and pushing the cerebellum caudally towards the 4th ventricle, and the cerebellar fold X against the medulla oblongata (Fig. 3I,J). Therefore, pressure build-up resulting from fluid accumulation in the lateral ventricle could affect the development of cerebellar folds I/II, III and X (Fig. 3J).

GFP fluorescence revealed Isl1-Cre activity in the superior colliculus, spinal cord, olfactory bulb, and clusters of cells in the olfactory tubercule, hypothalamus and medulla oblongata (Fig. 3K). Immunostaining revealed decreased H3K9me2 in nuclei of GFP-positive neuroepithelial cells lining the lateral ventricle in *G9a* homozygous compared to heterozygous mutants (Fig. 3L). This suggests that neuroepithelial cells derive from *Isl1*-expressing progenitors. Moreover, neuroepithelial cells were arranged in a thinner and disorganized layer in *G9a* mutants (Fig. 3L). Disrupting neuroepithelium integrity can compromise the production, flow or absorption of cerebrospinal fluid, ultimately leading to hydrocephalus (Feldner et al., 2017). Thus, G9a deficiency could affect neuroepithelium development and function. Hydrocephalus and hypoplastic cerebellum associated with hind limb paralysis suggest that *G9a* mutants model aspects of Dandy-Walker complex.

### G9a is required for growth of multiple fetal organs derived from *Isl1*-expressing progenitors

**Fig. 4.**
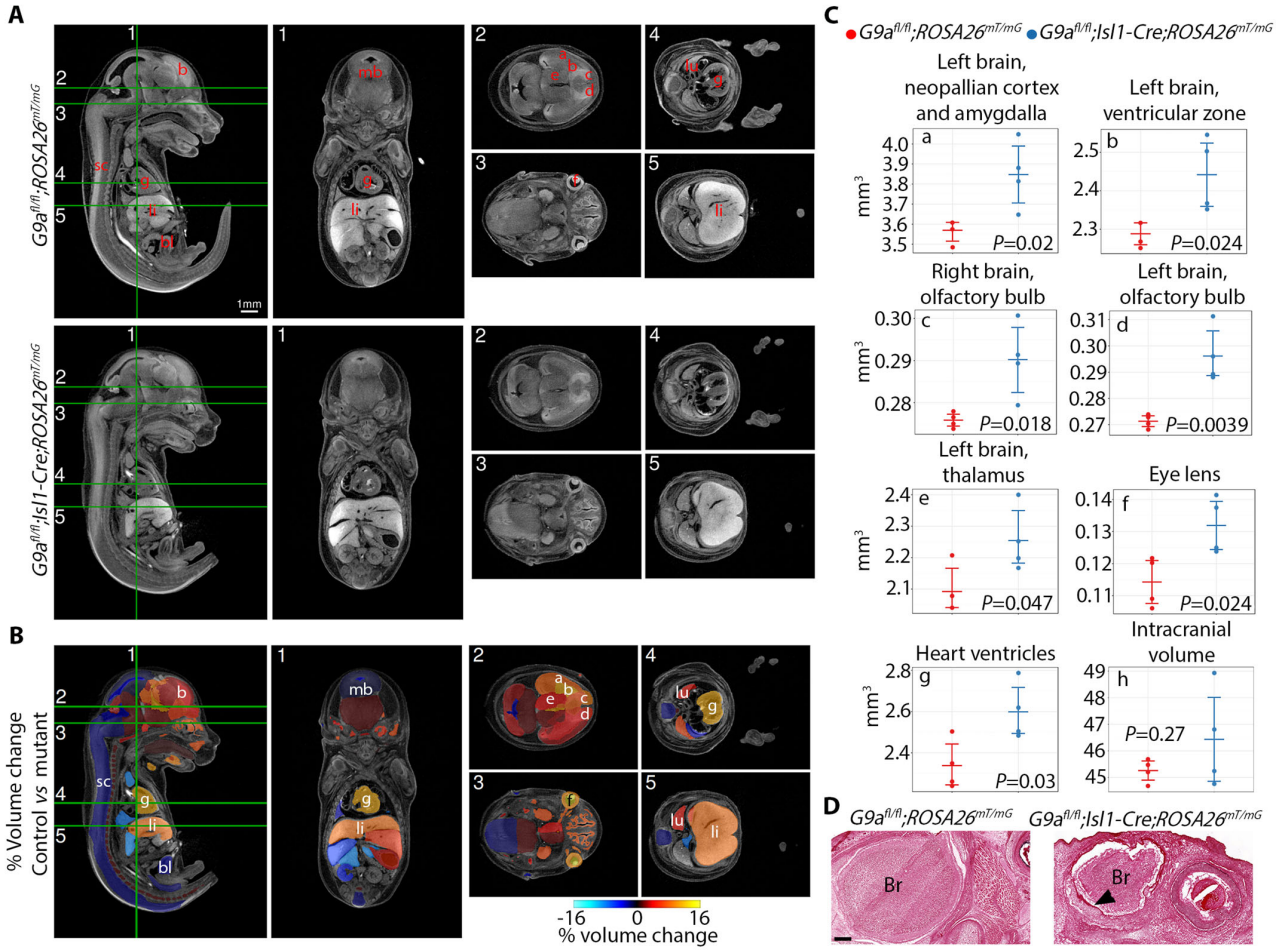
**G9a deficiency in *Isl1*-expressing progenitors controls growth of fetal brain structures.** (A) Sagittal and coronal isotropic sections of E15.5 control (*G9a^fl/fl^;ROSA26^mT/mG^*) and *G9a* mutant (*G9a^fl/fl^*;*Isl1-Cre;ROSA26^mT/mG^*) embryos. Numbers and green lines indicate section planes. Lower-case letters indicate the structures with significantly increased volume, quantified in C. (B) Images showing color-coded organ % volume change in mutant versus control embryos. mb, midbrain; sc, spinal cord; li, liver; bl, bladder; lu, lung. (C) Volumes for individual regions were calculated from reconstructions. Bars represent the mean and 95% confidence intervals. Volume comparisons were made via linear model with fixed effects for genotype. (D) Histological sections of control and mutant embryos showing the brain (Br) at the level of the eye. The arrowhead indicates a gap between the brain and the dura mater apparent in mutant embryos. Scale bar: 200 µm.

Dandy-Walker is a malformation that originates during fetal development. We assessed the requirement for G9a in development of organs derived from *Isl1*-expressing progenitors by X-ray computed tomography after iodine staining (Wong et al., 2013) in *G9a* mutant embryos versus controls at E15.5 (Fig. 4A-C). Analysis of 3D reconstructions revealed that the volume of the cerebellar primordium was comparable between mutant and control embryos (Table S1), suggesting that cerebellar growth is affected at a later stage. Instead, among 72 regions analyzed, the volume of derivatives of neuronal precursors in the brain was predominantly increased. Specifically, the volume of the left-brain neopallian cortex and amygdala, ventricular zone, left and right olfactory bulb, and left-brain thalamus were significantly increased in *G9a* mutant embryos (Fig. 4B,C). The volume of the lens of the eye, in which *Isl1* is expressed (Pan et al., 2008), and the heart ventricles was also increased. G9a inactivation in neural crest cells using Wnt1-Cre (Higashihori et al., 2017) or Sox9-Cre (Ideno et al., 2020) altered proliferation and differentiation of cranial bone cells and decreased ossification of the frontal bones, but did not cause hydrocephalus. Skull doming in *G9a^fl/fl^;Isl1-Cre* mice is likely secondary to hydrocephalus and not due to defective ossification because the thickness of the frontal bone was normal in mutant embryos (Fig. S2). Accordingly lineage tracing did not show *Isl1-*expressing progenitor contribution to the frontal or parietal bones (Zhuang et al., 2013). The intracranial volume trended to increase, and a gap was apparent between the brain and the dura mater in *G9a^fl/fl^;Isl1-Cre* embryos (Fig. 4C,D). This suggests that hydrocephalus might begin during embryonic development and that *Isl1*-expressing progenitors could be required for cerebrospinal fluid homeostasis. None of the organs analyzed had a smaller volume in mutants. These results suggest a function of G9a in limiting growth of neural crest progenitor derivatives.

## DISCUSSION

In this study, we found that inactivating G9a in mouse embryos using Isl1-Cre causes defects in the heart, nervous system and hind limbs. During the generation of experimental animals, we noticed progressive loss of these phenotypes in *G9a* mutants. Accordingly, we found that recombinase activity of the *Isl1-Cre* line was decreased (Fig. 1A), which can happen with an increased number of generations of Cre transgenic lines (Schulz et al., 2007). Inactivation of G9a using a freshly rederived *Isl1-Cre* line with high recombination efficiency (Fig. 1A) caused embryonic lethality between E14 and E17.5 (data not shown), preventing further analysis of postnatal phenotypes. This suggests that G9a in *Isl1*-expressing progenitor cells is essential for embryonic development. Thus, in this study, Isl1-Cre-mediated recombination bypassed embryonic lethality, revealing a requirement for G9a in the postnatal development of a subset of descendants of *Isl1*-expressing progenitors.

Deficient differentiation, expansion or migration of progenitors of the second heart field causes outflow tract and septation defects (Cai et al., 2003; Moretti et al., 2006). However, the outflow tract appeared normal, and the interventricular septum was properly formed in *G9a* mutants, suggesting that the second heart field developed normally. Instead, the volume of ventricles in the fetal heart was increased, and the postnatal heart and cardiomyocytes were hypertrophied in *G9a* mutants (Fig. 2). This suggests that G9a limits hypertrophic cardiomyocyte growth in both the adult (Papait et al., 2017) and fetal heart. Fetal heart overgrowth and hypertrophy of postnatal cardiomyocytes in the interventricular septum and left ventricle in *G9a* mutants suggest potential involvement of dysregulated paracrine signaling from progenitors or derivatives of the second heart field. G9a regulates signaling pathways that control cardiac morphogenesis and homeostasis. For example, G9a suppresses Wnt signaling in rhabdomyosarcoma (Pal et al., 2020), whereas it activates it in non-small cell lung cancer (Zhang et al., 2018). Wnt signaling from the first heart field is required for second heart field development (Miyamoto et al., 2023) and adverse myocardial remodeling (Bergmann, 2010). However, signaling between the first and second heart fields is still poorly understood. Uncovering G9a-controlled paracrine signaling mediators in *Isl1*-expressing progenitors and their cellular targets is required to test this possibility, and to elucidate the basis of cardiomyocyte hypertrophy and overgrowth of first heart field derivatives in *G9a* mutants. In the adult heart, G9a limits hypertrophy by mediating silencing of a pathological gene expression program (Papait et al., 2017). Our analysis does not rule out the possibility that hypertrophy of the postnatal heart might reflect adaptation to a pathological process. Intriguingly, the volume of fetal brain structures and the eye lens was also increased in *G9a* mutants, suggesting a broader function of G9a controlling growth of multiple organs during fetal development. This contrasts with an established function of G9a promoting cancer growth (Garcia-Dominguez et al., 2022; Haebe et al., 2021; Mabe et al., 2020), suggesting specific functions of G9a in growth of the heart and the developing brain.

Hydrocephalus, underdeveloped cerebellum and hind limb dysfunction in *G9a* mutant mice are reminiscent of Dandy-Walker complex, which is thought to originate from defective neural crest development (Coban et al., 2010; Squires et al., 1998). Our findings support this hypothesis. However, G9a dysfunction or mutations in its gene have not been linked to Dandy-Walker complex. Nonetheless, dysregulation of G9a downstream targets might be involved. For example, *FOXC1* and *FOXL2*, which are mutated in people affected by Dandy-Walker and regulate cerebellum development (Aldinger et al., 2009; Lim et al., 2011), are hypermethylated in mantle cell lymphomas with increased levels of G9A (Wang et al., 2021). Moreover, genes that regulate neural crest development and that are mutated in Dandy-Walker, were downregulated in *G9a* mutant descendants of *Isl1*-expressing progenitors (Fig. 1F). Most notably, *Foxc1* was highly expressed in wild type, but strongly downregulated in *G9a* mutant *Isl1*-expressing progenitors (Fig. 1F). *Foxc1* null mice have cerebellar vermis hypoplasia and foliation defects, modeling Dandy-Walker (Aldinger et al., 2009). A disarrayed neuroepithelium in the lateral ventricle of *G9a* mutants (Fig. 3L) suggests that altered cerebrospinal fluid dynamics could potentially cause hydrocephalus. Neuroepithelial defects have not been directly linked to Dandy-Walker. However, the ependymal cell layer was compromised in mice with severe cerebellum malformation due to mutation of platelet-derived growth factor C (*Pdgfc*) (Gillnas et al., 2022) and multiple PDZ domain protein (*Mpdz*), (Feldner et al., 2017; Yang et al., 2019), which was downregulated in *G9a* mutant neural crest descendants (Fig. 1F).

Stiffness and weakening of the leg muscles are associated with Dandy-Walker (Stoll et al., 1990). Limb paralysis in *G9a* mutants suggests potential involvement of altered development of neural crest *Isl1*-expressing progenitors. However, the hindlimb skeleton appeared normal in 4-week-old mutants. Alternatively, limb paralysis could be secondary to hydrocephalus, with ventricle dilation leading to oppression of the brain stem (Schmidt and Ondreka, 2019). Indeed, histological analysis suggests that lateral ventricle dilation could have pushed the cerebellum towards the medulla oblongata, which could in turn have pressed the brain stem down (Fig. 3J).

Identification of G9a targets in *Isl1*-expressing progenitors contributing to specific heart, brain, limb and eye structures, and revealing the cellular events affected upon its inactivation is required to uncover the mechanism of action of G9a. Our findings provide the basis of such studies and suggest that dysregulation of G9a-controlled gene expression might contribute to multicomponent diseases affecting the development of neural crest derivates.

## MATERIALS AND METHODS

### Mice

All procedures were approved by the Animal Care Committee at the Toronto Centre for Phenogenomics. The following mouse lines were used: *G9a^fl/fl^* (Sampath et al., 2007), *Isl1-Cre* (Yang et al., 2006) and *ROSA26^mT/mG^* (Muzumdar et al., 2007). Mice were bred in a C57B6 background, and maintained in vented cages under a 12-h dark-light cycle with *ad libitum* access to standard chow (Tekland Global 18% Protein Rodent Diet, ENVIGO, 491 TD.2918X) and water. Mice were genotyped using the primers listed in Table S2.

### Specimen imaging

Embryos at E10.5 and brains were dissected in cold PBS and immediately imaged to detect GFP. Brains were dissected in cold PBS and imaged immediately afterward. Fixed brains were cut sagittally and imaged. Specimens were imaged under a Nikon SMZ1500 stereo microscope. Images presented are representative of five specimens per genotype obtained from different litters.

### Immunofluorescence

Tissues were dissected in PBS and preserved by fixing them overnight at 4°C in 4% paraformaldehyde (PFA). They were then washed three times with PBS for 10 min at room temperature and left in 30% sucrose/PBS at 4°C until they sank to the bottom of the tube. Tissues embedded in O.C.T. Compound (Tissue-Tek) were then sliced into 4 μm frozen sections, which were mounted on glass slides. The slides were then fixed in 4% PFA for 5 min, followed by three washes with PBS for 5 min each. To prevent nonspecific binding, the slides were blocked in PBS with 5% goat serum and 0.1% Triton X-100 for 15 min, after which they were incubated overnight at 4°C with primary antibodies in a humidified chamber. After washing the slides three times with PBS for 10 min each, they were incubated with secondary antibodies diluted in blocking buffer for 1 h at room temperature. The slides were then washed three times with PBS for 5 min each and with PBS containing 0.05% Tween 20 for 5 min. Finally, the slides were mounted in Vectashield Mounting Medium with DAPI (Vector Laboratories). Antibodies used were GFP (GeneScript, A01694, 1:1000) and H3K9me2 (Cell Signaling Technology, 9753, 1/500). Immunostaining was performed in sections of three hearts or brains from mice of each phenotype and collected from different litters.

### Cell sorting and qPCR

GFP-positive cells were sorted from embryos incubated in TrypLE Express (Thermo Fisher Scientific, 12604-013) for 30 min at 37°C for cell dissociation. After centrifugation, the resulting pellets were then incubated in 1× Red Blood Cell Lysis Solution (MACS Miltenyi Biotec, 130-094-183) at room temperature for 10 min. Subsequently, the cells were resuspended in 250 µl Dulbecco's modified Eagle medium (Wisent, 319-005-CL) containing 1% fetal bovine serum, 1 mM EDTA and 2 µg/ml propidium iodide (Sigma-Aldrich, P4170), which aided in detecting dead cells. Finally, the cells were sorted using MoFlo-Astrios BYRV equipment. To generate cDNA, 10 ng total RNA was processed with a SuperScript VILO cDNA Synthesis Kit (Thermo Fisher Scientific). For qPCR, 10 pg cDNA was mixed with SsoAdvanced Universal SYBR Green Supermix (Bio-Rad). qPCR was run on a CFX384 Touch Real-Time PCR Detection System (Bio-Rad) using the primers listed in Table S2.

### Histology

Brains were dissected in PBS, fixed in 4% paraformaldehyde overnight at 4°C, and washed in PBS 3× for 10 min each at room temperature. Samples were processed for histology and stained with Hematoxylin and Eosin as previously described (Roy et al., 2018). Brains were sectioned sagittally and transversally, and imaged in a 3DHistech Pannoramic Flash II Slide Scanner. Images of histological sections presented are representative of three specimens per genotype; mice were from different litters. Quantification from histological sections was performed unaware of specimen genotype.

### Cardiomyocyte cross-sectional area

Hearts were dissected in PBS, incubated in 30% sucrose overnight at 4°C and embedded in O.C.T. Compound (Tissue-Tek) for cryosectioning. Then, 4 µm sections were stained with Wheat Germ Agglutinin, Alexa Fluor 594 Conjugate (Invitrogen, W11262) for 10 min and washed 3× for 10 min each with PBS. Sections were mounted with VECTASHIELD Antifade Mounting Medium with DAPI (Vector Laboratories) and imaged under a Nikon Eclipse Ni microscope. Cardiomyocyte cell surface area was quantified using ImageJ from images of the right and left ventricles. Fifty cardiomyocytes in three view fields of three different sections from each of six hearts per genotype were measured.

### Micro-computed tomography

E15.5 embryos were dissected and incubated in PBS (minus Ca/Mg) gently rocking for 10 min at 37°C and then washed twice with PBS before being fixed in 4% paraformaldehyde overnight at 4°C. Embryos were then stored in PBS with 0.02% sodium azide at 4°C until stabilization in hydrogel following the CLARITY method (Chung et al., 2013). Embryos were immersed in 50 ml of 0.1N iodine standard solution (Fisher Scientific, 1L,SI86-1) for 24 h on a rotator at room temperature and washed in 50 ml PBS for 1 h before embedding in 1% regular melting point agarose in 11 mm centrifuge tubes (Beckman Instruments, Palo Alto, CA, USA). Embryos were then imaged in a Bruker 1272 micro-computed tomography system with a 0.5 mm aluminum filter, 100 kV, 100 µA current, with 0.3° steps around the full embryo. Images were reconstructed at a resolution of 27 µm.

The embryo images were processed through a registration pipeline to produce an unbiased reference space and an average image representative of the sample (Friedel et al., 2014; Nieman et al., 2018). Each embryo image was also individually mapped to an existing embryo atlas using multiple automatically generated templates (Chakravarty et al., 2013). The segmentations resulting from this mapping were used for volumetric analysis. Statistical comparisons of volumes by genotype were completed using the R statistical computing package (https://www.r-project.org/) via a linear model with fixed effect terms for the intercept and for the difference between genotypes.

## Supplementary Material

10.1242/biolopen.059894_sup1 Supplementary information
