# Supplementary material for: G9a inactivation in progenitor cells with Isl1-Cre with reduced recombinase activity models aspects of Dandy-Walker complex
Source: Biol Open. 2023 Jul 28;12(8):bio059894. doi: 10.1242/bio.059894 (PMC10399207; doi:10.1242/bio.059894)
Supplement: Supplementary information [file biolopen-12-059894-s1.pdf]

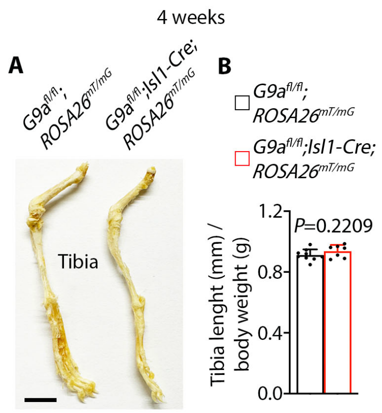

Fig. S1. Hind limb skeleton is normal in *G9a* mutants. (A) Hind limb skeleton of control (*G9a*$^{fl/fl}$;*ROSA26*$^{mT/mG}$) and *G9a* mutant (*G9a*$^{fl/fl}$;*Isl1-Cre;ROSA26*$^{mT/mG}$) mice at 4 weeks of age. (A) Tibia length to body weight ratio of control and mutant mice. Bars represent the mean +/- s.d. of 8 mice per genotype. Data was analyzed by paired two-tailed Student's t-test.

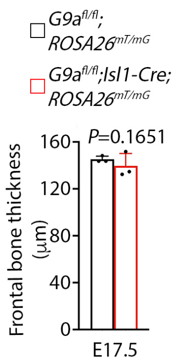

Fig. S2. Frontal bone thickness is normal in *G9a* mutants. Thickness of the frontal bone of control (*G9a*$^{fl/fl}$;*ROSA26*$^{mT/mG}$) and *G9a* mutant (*G9a*$^{fl/fl}$;*Isl1-Cre;ROSA26*$^{mT/mG}$) E17.5 embryos. Bars represent the mean +/- s.d. of 3 mice per genotype. Data was analyzed by paired two-tailed Student's t-test.

Biology Open • Supplementary information

Table S1. Volume in square micrometers of different embryonic regions measured from microcomputed tomography

Click here to download Table S1

Table S2. Primers

Primers used for genotyping the *G9a* “floxed” alle and the *Isl1tm1(cre)* transgenic line.

Target Gene	Forward	Reverse	Product length
G9a	CACGCTGCCTAGATGGAGCATGCC	GTGTGAGCCTGTGTTCTGGGGATTA	
WT allele: 368/ Floxed allele: 504			
Isl1tm1(cre)	GCCACTATTTGCCACCTAGC	AGGCAAATTTTGGTGTACGG	250

Primers for qPCR.

Target Gene	Forward	Reverse	Product length
Foxc1	CAACATCATGACGTCGCTGC	CCCGGAGAGTAGGCACCCA	135
Zic1	CCGGCTGAGGAGCCTTGAT	AGGCAAGAGAGATGACTGCCG	134
Zic4	GGCCCAGTGGGACATTGA	GGTCATTTCGGAGGTGGTGAAG	89
Mpdz	ACCAGCATATTACCGATTGAAGAAG	TGCTCACATGAGCCACCACTA	151
Fgf8	GCTCATTGTGGAGACCGATACTT	TGGCAATTAGCTTCCCCTTCT	101
Lamc1	CTGCCGCCAATGTGTCAATC	GCTTCTTCTGCCAAGAGGGT	76
Foxl2	GCTTACCTCTTGGCCCTCTC	CGCTTCTAGTCGCAGACAGT	90
Cip2a	GATTCCACCGCCTGCTTGAA	AGCTTTTGTCCAGAAACTACCTCC	119
Fgf17	GGCTTCTCTGGGACTCTACCTC	CGGGTGATTCTCCCCCTGTGTT	180
Rpl13a	TCCCTCCACCCTATGACAAG	GTCACTGCCTGGTACTTCC	136

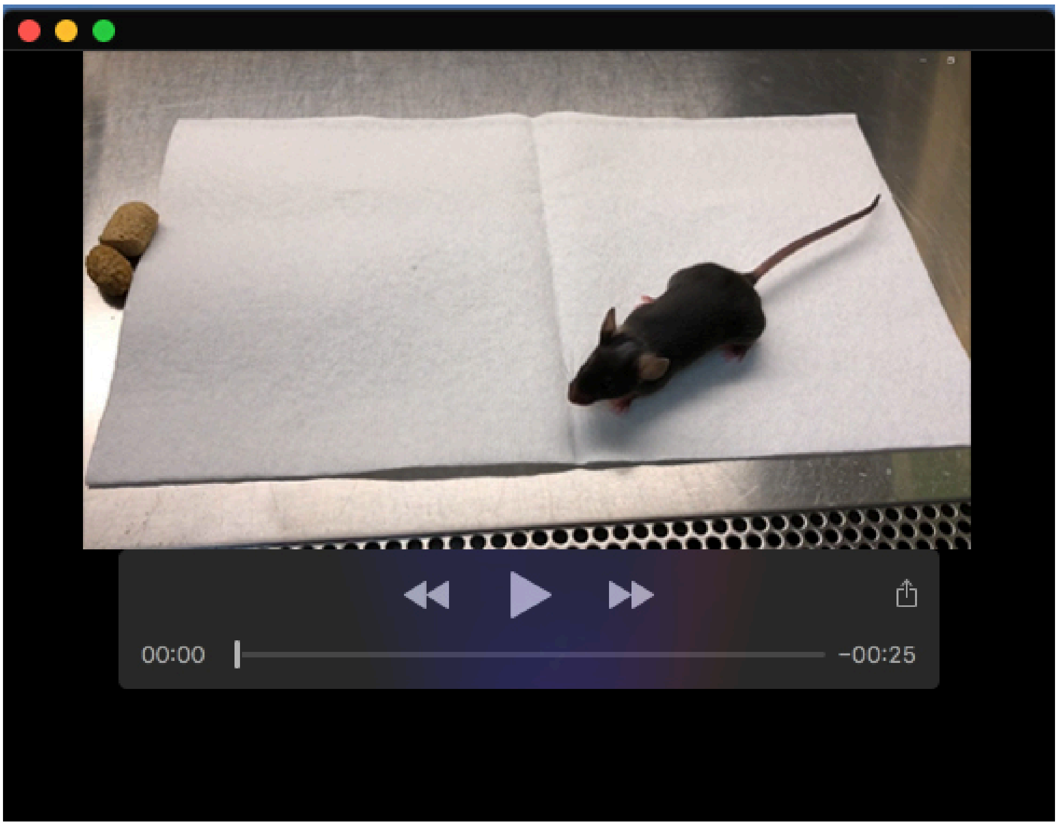

Movie 1. Control *G9a$^{fl/fl}$;ROSA26$^{mT/mG}$* mouse walking.

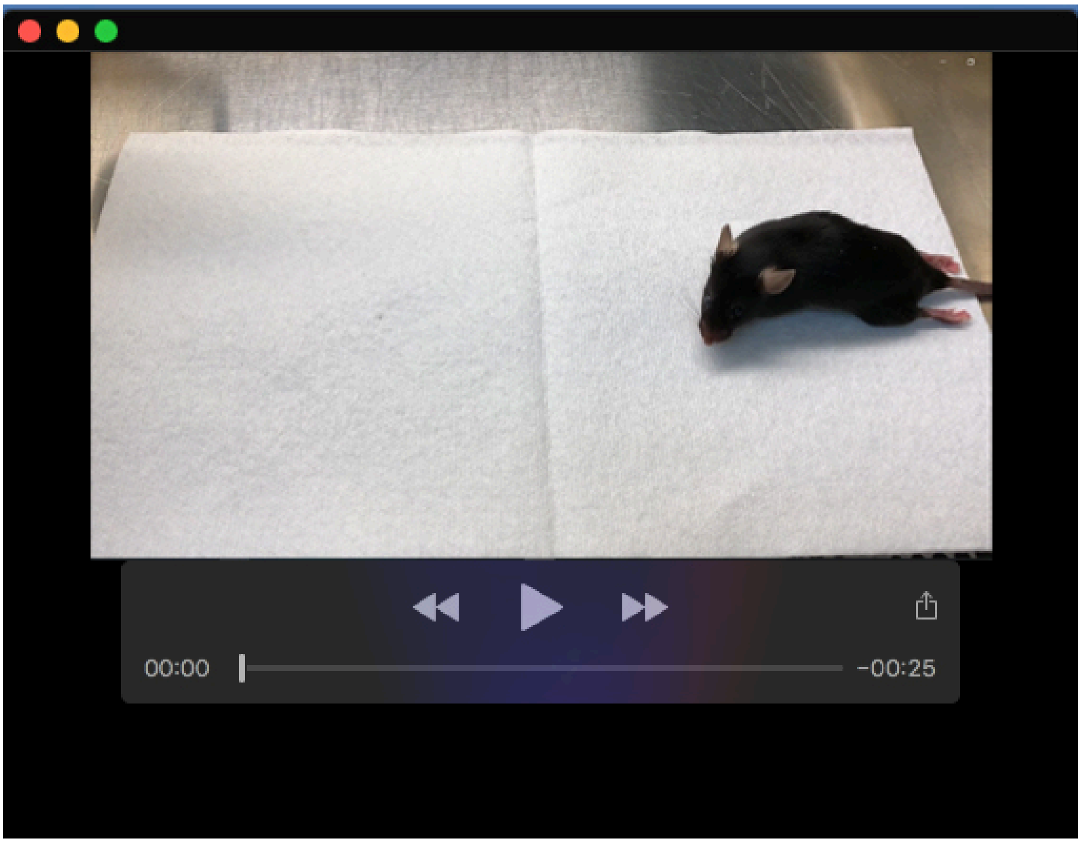

Movie 2. G9a mutant *G9a$^{fl/fl}$;Isl1-Cre;ROSA26$^{mT/mG}$* mouse walking.

Biology Open • Supplementary information
